# Micro electrical fields induced MSC-sEVs attenuate neuronal cell apoptosis by activating autophagy via lncRNA MALAT1/miR-22-3p/SIRT1/AMPK axis in spinal cord injury

**DOI:** 10.1186/s12951-023-02217-2

**Published:** 2023-11-27

**Authors:** Kewei Li, Zhong Liu, Peipei Wu, Shenyuan Chen, Min Wang, Wenhui Liu, Leilei Zhang, Song Guo, Yanbin Liu, Pengcheng Liu, Beiting Zhang, Lin Tao, Hua Ding, Hui Qian, Qiang Fu

**Affiliations:** 1 Department of Orthopedics, Shanghai General Hospital, Shanghai Jiao Tong University School of Medicine, Shanghai, 200080 China; 2 Key Laboratory of Laboratory Medicine of Jiangsu Province, School of Medicine, Jiangsu University, Zhenjiang, 212013 Jiangsu China; 3 Department of Laboratory Medicine, The First Affiliated Hospital of USTC, Division of Life Sciences and Medicine, University of Science and Technology of China, Hefei, 230001 Anhui China; 4 Department of Orthopaedics, Affiliated People’s Hospital of Jiangsu University, Zhenjiang, 212002 Jiangsu China; 5 Department of Orthopaedics, Dehong Hospital of Traditional Chinese Medicine, Dehong, 678400 Yunnan China

**Keywords:** Small extracellular vesicles, micro electrical field, Spinal cord injury, lncRNA MALAT1, Autophagy, Apoptosis

## Abstract

Spinal cord injury (SCI) is a traumatic condition of the central nervous system that causes paralysis of the limbs. Micro electric fields (EF) have been implicated in a novel therapeutic approach for nerve injury repair and regeneration, but the effects of human umbilical cord mesenchymal stem cell-derived small extracellular vesicles that are induced by micro electric fields (EF-sEVs) stimulation on SCI remain unknown. The aim of the present study was to investigate whether EF-sEVs have therapeutic effects a rat model of SCI. EF-sEVs and normally conditioned human umbilical cord mesenchymal stem cells-derived small extracellular vesicles (CON-sEVs) were collected and injected intralesionally into SCI model rats to evaluate the therapeutic effects. We detect the expression of candidate long noncoding RNA metastasis-associated lung adenocarcinoma transcript 1 (lncRNA-MALAT1) in EF-sEVs and CON-sEVs. The targets and downstream effectors of lncRNA-MALAT1 were investigated using luciferase reporter assays. Using both in vivo and in vitro experiments, we demonstrated that EF-sEVs increased autophagy and decreased apoptosis after SCI, which promoted the recovery of motor function. We further confirmed that the neuroprotective effects of EF-sEVs in vitro and in vivo correlated with the presence of encapsulated lncRNA-MALAT1 in sEVs. lncRNA-MALAT1 targeted miR-22-3p via sponging, reducing miR-22-3p’s suppressive effects on its target, SIRT1, and this translated into AMPK phosphorylation and increased levels of the antiapoptotic protein Bcl-2. Collectively, the present study identified that the lncRNA-MALAT1 in EF-sEVs plays a neuroprotective role via the miRNA-22-3p/SIRT1/AMPK axis and offers a fresh perspective and a potential therapeutic approach using sEVs to improve SCI.

**Supplementary Information:**

The online version contains supplementary material available at 10.1186/s12951-023-02217-2.

## Introduction

Spinal cord injury (SCI) results in severe trauma to the central nervous system that can lead to severe neurological dysfunction, including paralysis, incontinence, and chronic pain, with high disability and high mortality [1]. A large number of studies have shown that SCI is often accompanied by complex pathological and physiological changes, mainly including the two stages of primary and secondary injury, where secondary injury plays a key role in the recovery of neurological function after SCI [2, 3]. Primary injury refers to the irreversible destruction of spinal cord tissue as a direct result of direct or indirect violence. Secondary injury specifically includes oxidative stress, the inflammatory response, ischemia, apoptosis, and edema, significantly affecting patient prognosis after SCI [4, 5]. Although the exact molecular pathways of secondary injury remain elusive, apoptosis plays a particularly important role. Accumulating evidence has demonstrated that inhibiting apoptosis can lead to improvement of patient prognoses [6, 7]. Thus, attenuating or blocking apoptosis may be a promising therapeutic strategy for SCI and benefit patient recovery suffered with SCI.

In recent years, more studies focused on the role of autophagy in SCI [8, 9]. Autophagy is an important defensive and protective mechanism of the body to maintain metabolism and homeostasis by eliminating protein aggregates and damaged organelle; autophagy can regulate homeostasis in physiological and pathophysiological environments and participate in the occurrence and development of central nervous system diseases [10]. Many studies have confirmed that autophagy plays an important role in SCI, and activating autophagy can inhibit apoptosis, leading to neuroprotective effects in SCI [11, 12]. Autophagy has been identified as a therapeutic and intervention target for a variety of diseases, including SCI [13]. Previous studies have reported that treatment with curcumin can promote the recovery of spinal cord function in SCI model rats by inhibiting Akt/mTOR signaling to promote autophagy, reduce neuronal apoptosis, improve spinal cord integrity, and inhibit the inflammatory response [14]. Therefore, the regulation of autophagy has profound implications for the treatment of SCI.

In addition, mesenchymal stem cell-derived small extracellular vesicles (MSC-sEVs) have attracted much attention in SCI therapy. Small extracellular vesicles (sEVs) are microvesicles around 30–200 nm in diameter that are secreted by various cell types with low immunogenicity, stable circulation in the body, and nanometer size, allowing them to cross the blood–brain barrier to exert significant therapeutic effects in SCI [15, 16]. Several studies have confirmed that MSC-derived sEVs have multiple positive effects on the regeneration of injured spinal cord tissue and that they can repair SCI by promoting axon growth, regulating the inflammatory response, activating autophagy, and inhibiting apoptosis [17–19]. They are, therefore, considered to be a promising cell-free therapy for SCI repair.

Micro electric fields (EF) are used as a physical method that can be used to regulate various cellular processes such as cell proliferation, differentiation, and apoptosis [20]. Endogenous EF play an indispensable role in many biological processes including embryonic development and injury repair [21, 22]. EF are widely used to treat essential tremor in Parkinson’s disease, pelvic floor dysfunction, vagus nerve electrical stimulation in epilepsy, and other common clinical diseases due to its safety, lack of immune response, simple implementation, and controllable parameters, with good clinical efficacy [23, 24]. A previous study demonstrated that three patients with SCI were able to walk again after receiving directional EF stimulation, providing a novel therapy to induce the recovery of neurological function after SCI and bringing hope to patients who are paralyzed after SCI [25]. Although the potential benefits of EF stimulation after SCI are clear, the cellular and molecular mechanisms driving these functional improvements remain elusive. Moreover, sEVs play important roles in intercellular communication and paracrine signaling, and a recent study reported that EF can promote the secretion of sEVs by cells [26]. Therefore, EF may increase the levels of functional molecules such as nucleic acids and proteins in sEVs, stimulating their corresponding physiological functions. However, the effects of human umbilical cord MSC (hucMSC) derived sEVs induced by micro electric fields (EF-sEVs) on SCI remain unknown.

In this study, we first investigated the protective roles of EF-sEVs using a rat model of SCI. EF-sEVs were enriched with the long noncoding RNA (lncRNA) metastasis-associated lung adenocarcinoma transcript 1 (lncRNA-MALAT1) that increases autophagy and exhibits antiapoptotic effects in vivo and in vitro, promoting nerve tissue repair and the recovery of motor function. We revealed that knockdown of lncRNA-MALAT1 in EF-sEVs significantly promoted neuronal cell apoptosis, and it was suggested that this may involve the miR-22-3p/SIRT1/AMPK signaling pathway.

## Materials and methods

### SCI model

Eight-week-old female Sprague–Dawley rats were purchased from the Animal Center of Jiangsu University, China. All rats were housed and handled in compliance with the regulations of the Animal Committee at Jiangsu University. Rats were anesthetized via isoflurane inhalation and subjected to laminectomy to expose the spinal cord at the level of the T9–T10 vertebrae. Then, an impactor (weighing 5 g, 2 mm in diameter, 150 mm in length) was vertically dropped onto the exposed spinal cord; the wound was then sutured. SCI was considered to be successfully induced when it resulted in spinal cord compression, swaying legs, tail swing reflexes, and slow paralysis. All rats were housed after surgery in a separate environment at 24 °C with adequate water, food, and clean bedding, and assistance for urination was provided three times daily. Rats were randomly assigned to four groups and, immediately after laminectomy and SCI operation, received an intralesional injection of normally conditioned sEVs (CON-sEVs), EF-sEVs, or an equal volume of PBS using a microneedle. Specifically, when the spinal cord was contused, immediately after hemostasis, 80 µL of either PBS or PBS containing CON-sEVs or EF-sEVs (2.4 mg total protein, Bicinchoninic acid assay) were slowly injected into the upper and lower sides of the spinal cord lesion site with a depth of 0.9 mm using a pulled-glass micropipette. After the solution was absorbed, the musculature and skin were sutured sequentially. Rats receiving laminectomy but no spinal cord compression were prepared as a sham group.

### Functional behavioral assessments

Neurological function was evaluated using the Basso, Beattie, and Bresnahan (BBB) locomotor rating scale at 1, 7, 14, 21, and 28 days after surgery. In brief, BBB scores range from 0 points (complete paralysis) to 21 points (normal locomotion). Footprint analysis was used to evaluate the recovery of hindlimb muscle strength and motor coordination. The fore- and hindlimbs of the rats were dipped into black and red ink, and the rats walked on a narrow passage covered with paper. The distance between the left and right rear paws was measured and considered the base of support. Stride length was measured as the perpendicular distance between the fore and hind limbs to assess the coordination ability.

### Histological analysis

Rats were deep euthanized using 0.6% sodium pentobarbital (10 g/0.1 mL) 4 weeks after SCI. Animals were then intracardially perfused with PBS followed by 4% paraformaldehyde. The spinal cord containing the injury site was dissected, fixed in 4% paraformaldehyde for 24 h, and embedded in paraffin. Samples were then cut into 20 μm sections. A general review and lesion cavity assessment was performed using hematoxylin and eosin (HE) staining in samples from each spinal cord treatment group. Nissl staining was used to estimate the number of neurons at the lesion sites.

### Immunofluorescence staining assays

Rat spinal cords (postoperative week 4) were embedded in paraffin and sectioned, and the sections were dehydrated and treated to inhibit peroxidase activity. Next, the sections were blocked using 5% bovine serum albumin and 0.3% Triton X-100 and incubated overnight at 4 °C with primary antibodies. The primary antibodies were anti-neurofilament (NF;Abcam, Cambridge, UK), anti-choline acetyltransferase (ChAT; Omnimabs, Alambra, CA, USA), and anti-glial fibrillary acidic protein (GFAP; Boster Bio, Pleasanton, CA, USA). After standard washing procedures, samples were incubated with secondary fluorescence-conjugated antibodies (Invitrogen, Waltham, MA, USA), and Hoechst (1:300; Sigma-Aldrich, St. Louis, MO, USA) was used to label cell nuclei. Images were captured using a microscope (Nikon, Tokyo, Japan).

### Cell culture and induction of EF

hucMSCs were obtained and cultured as previously reported [27]. In brief, fresh umbilical cord tissue was acquired from patients in the Affiliated Hospital of Jiangsu University after written informed consent was obtained. The use of human umbilical cord samples was approved by the ethics committee of Jiangsu University. hucMSCs were maintained in 10% fetal bovine serum (FBS; ExCell Bio, Shanghai, China) and Dulbecco’s Modified Eagle Medium. hucMSCs from passages 3–5 were used for further experiments. We designed an novel EF device in vitro for delivering direct current electrical stimulation to cultivated cells (Additional file 1: Fig. S1) and was manufactured by Changzhou Ruishen’an Medical Equipment Co., LTD. The hucMSCs were cultured under a 100 mV/mm electric field intensity 1 h per day for three consecutive days. Our previous study have determined that the optimal stimulus parameter was 100 mV/mm 1 h per day. PC12 cells were purchased from Procell Life Science & Technology Co., Ltd. (Wuhan, China) and maintained in 10% FBS (Excell Bio, China) and RPMI 1640 medium (Invitrogen, USA).

### sEVs isolation and identification

sEVs were extracted using ultracentrifugation as previously described [27]. The culture medium of the hucMSCs was replaced with sEVs-depleted 10% FBS, with or without a 100 mV/mm microcurrent, for an additional 3 days. When the confluency of the hucMSCs reached 90–100%, the two supernatants were collected and the both types of sEVs were extracted using standard methods, labeled as CON-sEVs and EF-sEVs, respectively. The morphology and size of the CON-sEVs and EF-sEVs were identified using transmission electron microscopy (FEI Tecnai 12; Philips, Amsterdam, Netherlands) and nanosight tracking analysis (NTA; Particle Metrix, Ammersee, Germany). The protein content in sEVs was measured using a Pierce Bicinchoninic Acid Protein Assay Kit (Thermo Fisher Scientific). Western blot analysis was used to identify sEVs surface markers including Alix, CD81, CD63, TSG101, and calnexin. For sEVs uptake experiments, CON-sEVs and EF-sEVs were incubated with DiI dye solution for 30–60 min at 37 °C in darkness. Labeled CON-sEVs and EF-sEVs were incubated separately with PC12 cells and imaged using confocal microscopy (Beckman Coulter, USA).

### RNA sequencing

For lncRNA-seq experiments, total RNA was extracted from sEVs using the miRNeasy Serum/Plasma Kit (Qiagen, Hilden, Germany) following the manufacturer’s instructions and RNA integrity numbers were analyzed using an Agilent 4200 TapeStation (Agilent Technologies, Santa Clara, CA, USA). Qualified total RNA was further purified using the RNA Clean XP Kit (Cat#A63987; Beckman Coulter, Inc., Brea, CA, USA) and RNase-Free DNase Set (Cat#79254, Qiagen). Purified total RNA was subjected to rRNA removal, fragmentation, first-strand cDNA synthesis, second-strand cDNA synthesis, end repair, 3′ end addition, ligation, and enrichment to complete the library construction of sequencing samples. The Illumina NovaSeq6000 sequencer was used, and the PE150 mode was selected for sequencing.

### Quantitative real-time PCR

Total RNA was extracted from cells and sEVs using TRIzol (Invitrogen, USA), and cDNA was synthesized using a reverse transcription kit (Nanjing Vazyme Biotech Co, Nanjing, China) with total RNA for mixing with SYBR-Green reagents to perform qRT-PCR experiments. Expression levels were evaluated using the 2^−ΔΔCT^ method. The sequences of primers were listed in Additional file 2: Table S1.

### Oligonucleotide transfection and lentivirus transduction

MiR-22-3p mimics, mimics NC, miR-22-3p inhibitor, and inhibitor NC were synthesized and purified by GenePharma (Suzhou, China). Transfection was performed using Lipofectamine 2000 (Invitrogen, USA). After transfection for 30 h, total RNA was isolated from PC12 cells. Lentiviral constructs for short hairpin MALAT1 (shMALAT1) were generated by Hanbio (Shanghai, China). Scrambled lentiviral constructs were used as negative controls. The sequences of the shRNA were listed in Additional file 2: Table S2.

### Establishment of an in vitro model of SCI

The PC12 cell line, derived from pheochromocytoma of *Rattus norvegicus*, is commonly used to study nervous system diseases. PC12 cells have strong viability, and it has been previously confirmed that PC12 cells exposed to hydrogen peroxide (H_2_O_2_) can effectively replicate the effects of SCI in vitro [28]. This cellular SCI model is often used in in vitro studies of SCI. Therefore, the PC12 cells were exposed to 200µM H_2_O_2_ solution in Dulbecco’s Modified Eagle Medium without FBS for 24 h to develop the H_2_O_2_-induced model of oxidative stress.

### Flow cytometry

The rate of apoptosis was examined using flow cytometry. H_2_O_2_-induced PC12 cells were co-cultured with sEVs for 24 h, and cells were stained using the Annexin V-Fluorescein Isothiocyanate and Propidium Iodide Apoptosis Kit (Nanjing Vazyme Biotech Co, China). Cellular apoptosis rates were then estimated using flow cytometry (BD, FACSCalibur, USA).

### Reactive oxygen species (ROS) measurement

To evaluate the protective function of sEVs for PC12 cells against oxidative stress, we performed a range of experiments. PC12 cells were seeded on a 12well plate and co-cultured with different sEVs. Subsequently, the culture medium was replaced with peroxidative medium containing 200 µM H_2_O_2_ for 24 h. Following the peroxidative culture, reactive oxygen species (ROS) levels were measured using an ROS assay kit (Beijing Solarbio Science & Technology Co., Ltd.) in accordance with the manufacturer’s protocols. Samples were imaged using fluorescence microscopy (Olympus, Tokyo, Japan), and relative fluorescence was calculated using ImageJ and GraphPad Prism 8.0 (GraphPad Software, San Diego, CA, USA) software.

### Western blot

Cells and spinal cord tissue were lysed using radio immunoprecipitation assay buffer (Thermo Fisher Scientific). Equal amounts of proteins were separated using sodium dodecyl sulfate-polyacrylamide gel electrophoresis gel and then transferred to polyvinylidene difluoride membranes, blocked with 5% bovine serum albumin, and incubated overnight at 4 °C with primary antibodies against p-AMPK (1:1000; Bioworld, USA), cleaved caspase 3 (1:1000; Cell Signal Technology, Danvers, MA, USA), Bcl-2 (1:1000; Abcam, UK), Bax (1:1000; Abcam, UK), beclin-1 (1:1000; Abcam, UK), LC3B (1:1000; Abcam, UK), P62 (1:1000; Abcam, UK), SIRT1 (1:1000; Abcam, UK), TSG101 (1:1000; Abcam, UK), CD63 (1:1000; Abcam, UK), CD81 (1:1000; Abcam, UK), β-actin (1:1000; Abcam, UK). The next day, the membranes were incubated with secondary antibodies (1:10,000; Jackson ImmunoResearch Labs, West Grove, PA, USA). Protein bands were detected using an ImageQuant LAS4000 mini chemiluminescence imager (GE Healthcare, Chicago, IL, USA).

### Luciferase assay

The MALAT1 mimic or MALAT1 inhibitor luciferase reporter plasmids and miR-22-3p mimics or miR-22-3p inhibitors were co-transfected into HEK293T cells together with the Renilla luciferase gene. Six hours following transfection, cells were cultured in complete medium for 18 h. Cells were then lysed, and luciferase activity was assessed using an enhanced luciferase assay kit (Nanjing Vazyme Biotech Co) following the manufacturer’s instructions. Luciferase activity levels were normalized to Renilla activity levels. Then, SIRT1 mimic or SIRT1 inhibitor luciferase reporter plasmids and miR-22-3p mimics or miR-22-3p inhibitors were also detected as described above. All plasmids and oligonucleotides were synthesized by Genepharma, and their sequences and modifications are shown in Additional file 2: Table S1.

### Statistical analysis

All data represent at least three independent replicates. Statistical analysis was performed using GraphPad Prism 8 (GraphPad Software). Data are presented as mean ± standard deviation (SD). The independent samples Student’s *t*-test was used to analyze differences between two unpaired groups, and differences between multiple groups were analyzed using one-way analysis of variance. *P*-values < 0.05 were considered statistically significant.

## Results

### Identification of sEVs derived from hucMSCs cultured under normal and EF conditions

**Fig. 1:**
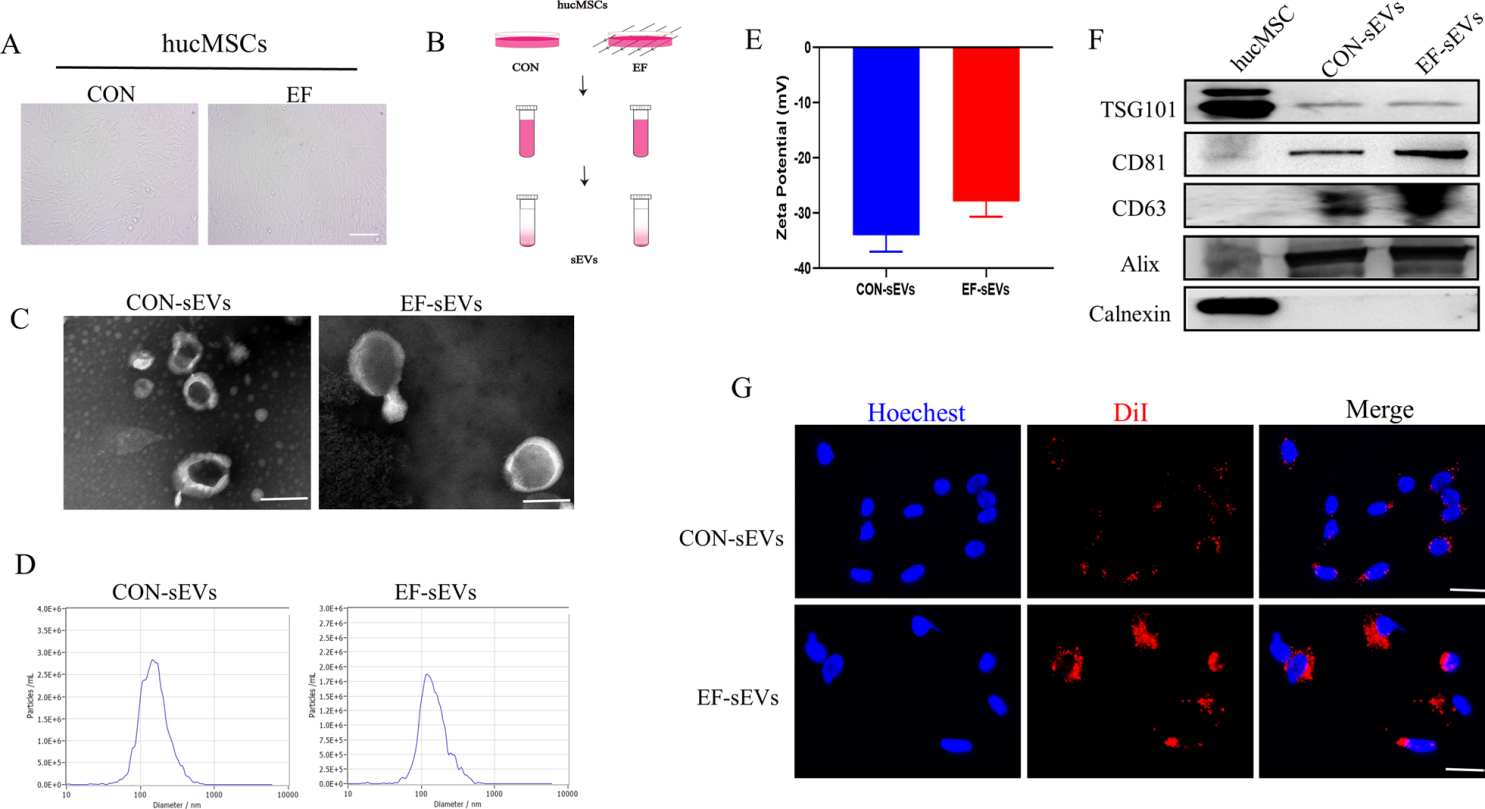
Identification of sEVs derived from hucMSCs cultured under normal and EF conditions. **A** Representative image showing the morphology of normal and EF-hucMSCs under light microscopy. **B** sEVs were extracted from hucMSCs supernatants under normal and EF conditions. **C** Transmission electron microscopy images of CON-sEVs and EF-sEVs. Scale bar = 200 nm. **D** NTA analysis of CON-sEVs and EF-sEVs revealed that sEVs from the two groups exhibited similar size ranges (50–180 nm). **E** Zeta potential of CON-sEVs and EF-sEVs. **F** Western blot showing positivity for sEVs markers, including TSG101, CD81, CD63, and Alix, and negativity for calnexin. **G** Uptake DiI-labeled CON-sEVs and EF-sEVs into PC12 cells. Scale bar =100 nm

hucMSCs were obtained and cultured, as described above. The identification and characterization of hucMSCs by related experiments confirmed that hucMSCs were successfully isolated and purified (Additional file 1: Fig. S2). Subsequently, hucMSCs were cultured under normal and EF conditions. Supernatants were collected and sEVs were extracted, labeled as CON-sEVs and EF-sEVs, respectively (Fig. 1A, B). Transmission electron microscopy showed a similar cup-shaped or spherical morphology of CON-sEVs and EF-sEVs (Fig. 1C). Nanoparticle tracking analysis confirmed that the particle size and zeta potential of CON-sEVs and EF-sEVs were similar (Fig. 1D, E). Western blot revealed positivity of both sEVs for the surface markers TSG101, CD81, CD63, and Alix, and they were negative for calnexin (Fig. 1F). In summary, these results confirmed that sEVs had been successfully isolated from hucMSCs. As shown in Fig. 1G, CON-sEVs and EF-sEVs were scattered around the nuclei of PC12 cells, and EF-sEVs were more abundant, indicating that sEVs could be phagocytosed normally by PC12 cells and further exhibit other forms of functionality and suggesting that EF-sEVs were more readily taken up by PC12 cells.

### EF-sEVs promoted better functional behavioral recovery after SCI in rats

**Fig. 2:**
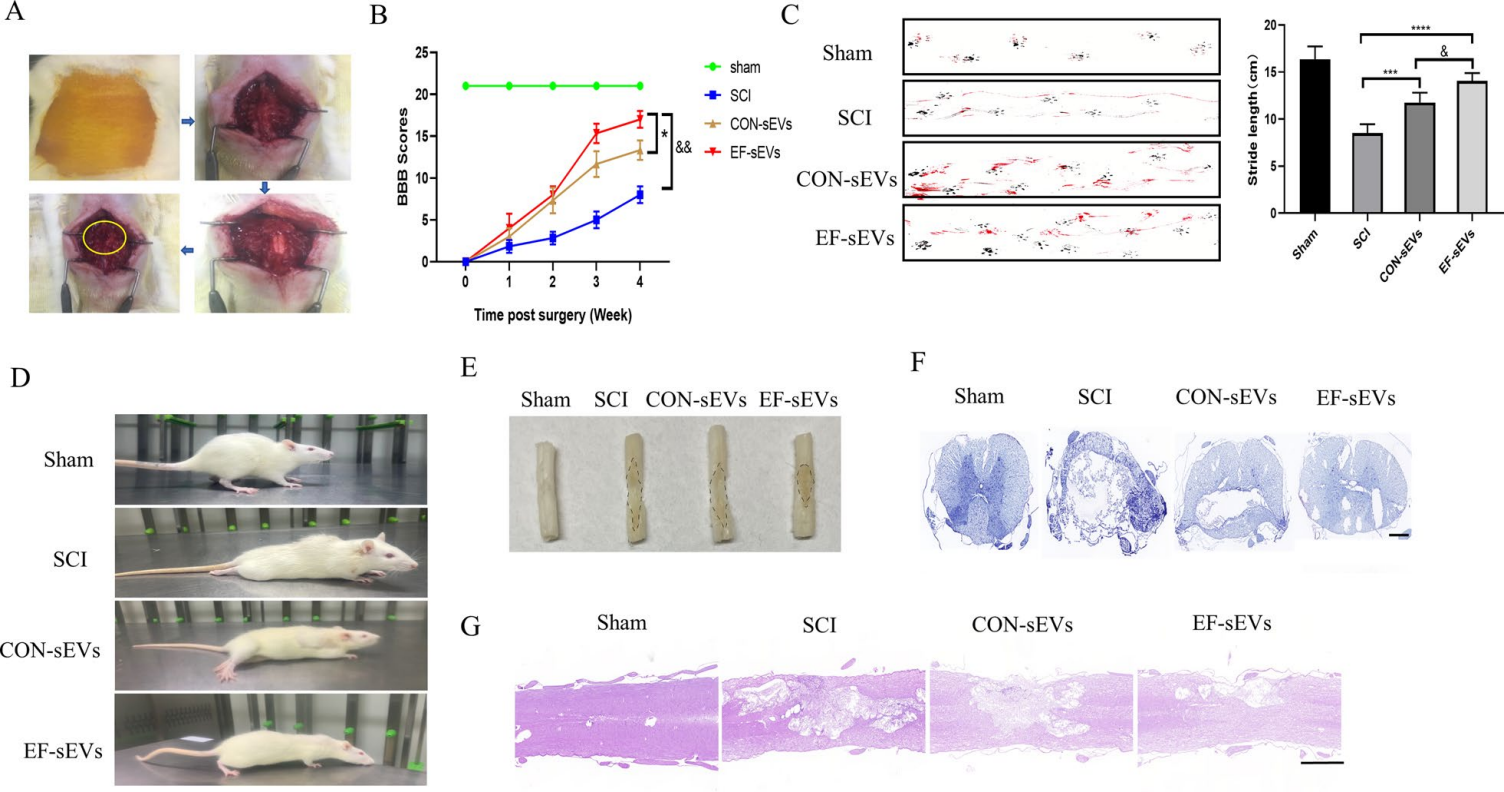
EF-sEVs promoted better functional behavioral recovery after SCI in rats. **A** The surgical processes and spinal cord compression. **B** Basso, Beattie, and Bresnahan (BBB) limb function scores at different times after SCI. **C** Representative footprints of an animal walking 4 weeks after SCI. Stride length analysis (right) of different groups. Black: front paw print; red: rear paw print. **D** Hindlimb vertical standing in rats after a 4-week recovery period postinjury. **E** Gross morphology of spinal sections; the area in the dotted line represents the extent of the scar. **F** Representative Nissl stainings of coronal sections of the spinal cord in different groups. Scar bar = 100 μm. **G** Representative HE images of spinal cord longitudinal sections 4 weeks postinjury in different groups. Scar bar = 500 μm. Statistical analysis was performed using a one-way analysis of variance. *P < 0.05, ***P < 0.001

hucMSC-sEVs have been widely shown to promote recovery from SCI, and the present study was designed to investigate whether EF-sEVs have more potent effects. EF-sEVs were used for SCI treatment in a rat model of SCI. The surgical processes is illustrated in Fig. 2A, including sterilization of the surgical area, incision of the skin muscle layer by layer, the complete exposure of the spinal cord after laminectomy, and impingement of the spinal cord. Over four consecutive weeks of evaluation after SCI induction, the EF-sEVs group had better recovery regarding rear paw placement and hindlimb movement according to BBB scores (Fig. 2B). The footprint test showed longer stride lengths in the EF-sEVs group, indicating sustained coordinated fore- and hindlimb movements (Fig. 2C). After 4 weeks of recovery, the EF-sEVs group could stand continuously on their rear paws and keep their trunk stable with their tails up (Fig. 2D). Spinal cord specimens showed less extensive lesions in the EF-sEVs group (Fig. 2E). Nissl staining showed a greater number of neurons in the EF-sEVs group compared with the SCI group (Fig. 2F). Furthermore, we analyzed histological morphology and the cavity area in injured spinal cords using HE staining 4 weeks after SCI (Fig. 2G). We observed severely damaged tissue and obvious cystic cavitations in the injured spinal cords. Following treatment with either CON-sEVs or EF-sEVs, the lesion areas were notably smaller than those in the SCI group. These results also indicated that the lesion area in the EF-sEVs group was significantly smaller than that in the CON-sEVs group.

### EF-sEVs promoted nerve tissue regeneration after SCI

**Fig. 3:**
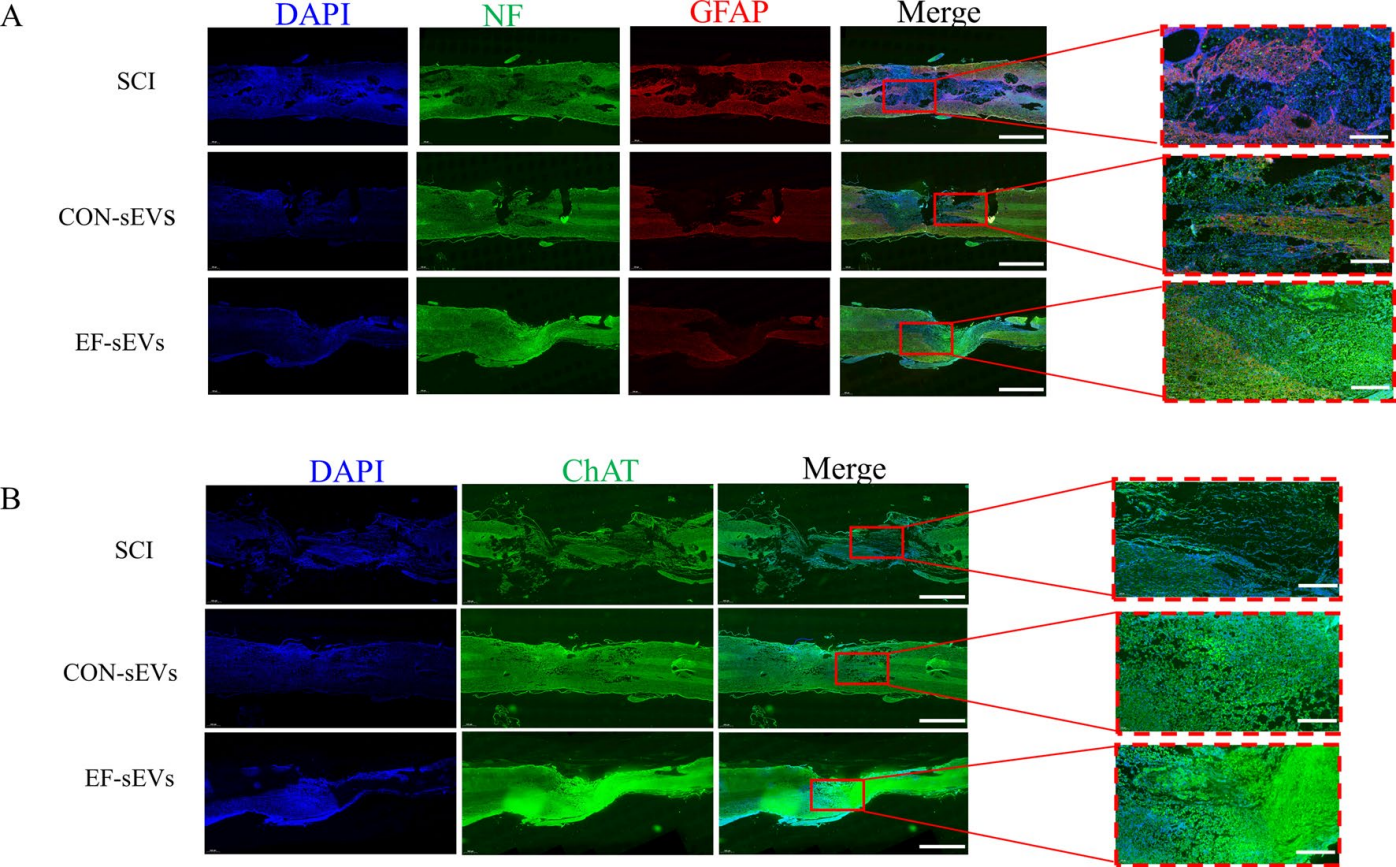
EF-sEVs promoted neurogenesis at the injury site in the spinal cord. **A** Representative immunofluorescence images showing the staining of neurofilaments (NF, green) and glial fibrillary acidic protein (GFAP, red) in lesion sites in different groups. **B** Fluorescent immunostaining of choline acetyl transferase (ChAT, green) in lesion sites in different groups. Scale bar=500 μm (**A**, **B** left line before magnification) and 100 μm (**A**, **B** right lines after magnification)

To further provide anatomical evidence to support the behavioral recovery, we examined nerve regeneration in the area of injury, looking at the distribution of NF and GFAP-positive astrocytes, which are the main components of glial scars. As is shown in Fig. 3A, in the SCI group, substantial GFAP expression and fewer NF expression in the injury sites 4 weeks after surgery. In contrast, the mean fluorescence intensity ratio of GFAP to NF in the injury sites of the EF-sEVs group were significantly reversed. In comparison to the CON-sEVs group, the EF-sEVs group showed a superior effect in terms of nerve fiber restoration, with a higher neuro-filament redistribution and a higher ratio of NF-positive to GFAP-positive cells. In addition, ChAT expression in spinal cord tissue was also assessed. The results of ChAT staining demonstrated that the ChAT delivery was significantly restored in the EF-sEVs group compared with the SCI group (Fig. 3B). The above evaluation results were consistent with the results from motor function recovery analysis and demonstrated the function of EF-sEVs in promoting nerve regeneration in SCI.

### EF-sEVs induced autophagy and inhibited apoptosis in vivo

**Fig. 4:**
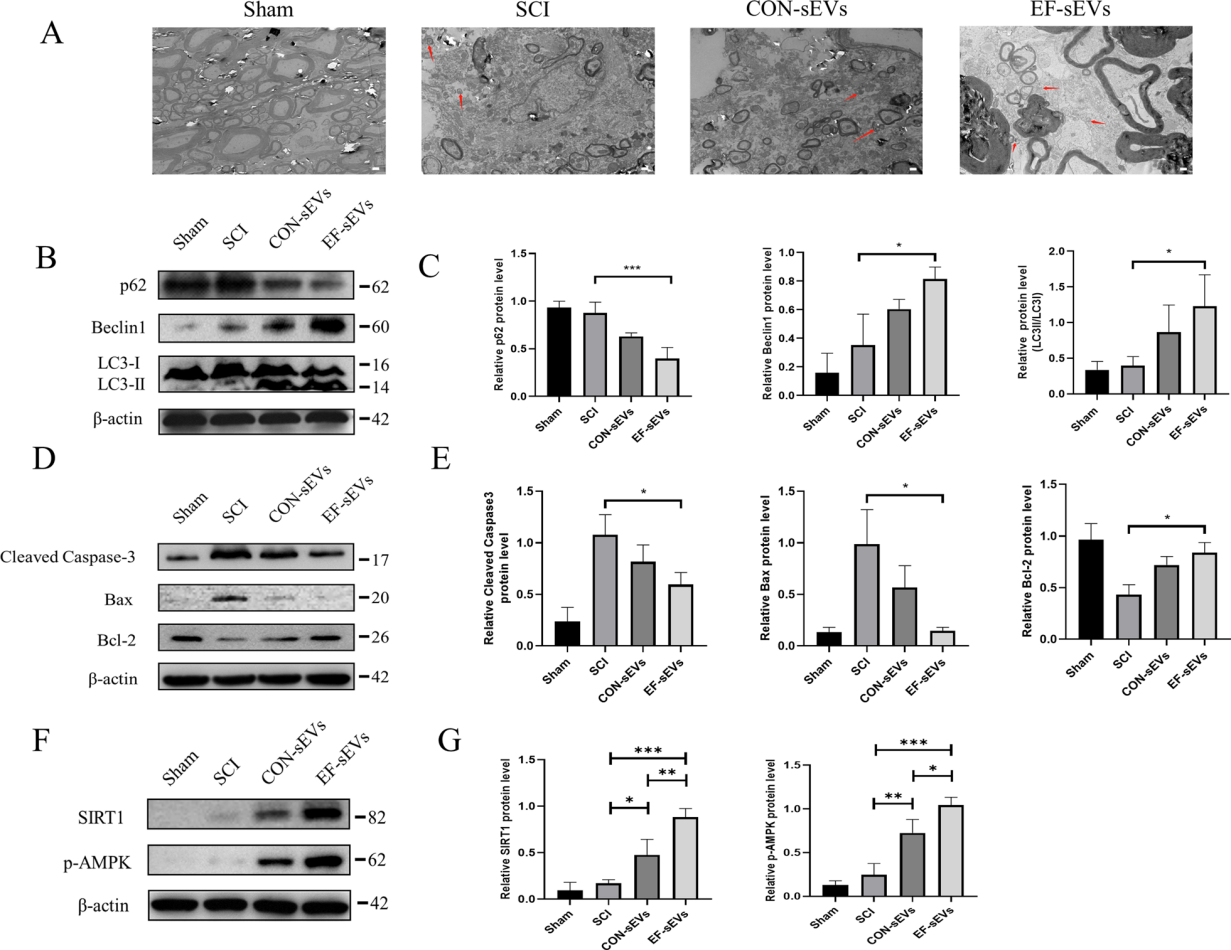
EF-sEVs induced autophagy and inhibit apoptosis in vivo. **A** Ultrastructural images of spinal cord cells 4 weeks postinjury; red arrows represent autophagosomes. Scale bar=5 μm. **B** Western blot analysis of autophagy-related proteins, including Beclin1, p62, and LC3II/LC3I 4 weeks post-injury. **C** Semiquantitative analysis of autophagy-related protein levels. **D** Western blot analysis of apoptosis-related proteins including Bax, Bcl-2, and cleaved caspase 3 4 weeks post-injury. **E** Semiquantitative analysis of apoptosis-related protein levels. **F** Western blot analysis of SIRT1 and p-AMPK protein expression 4 weeks postoperatively. **G** Semiquantitative analysis of SIRT1 and p-AMPK protein expression levels. Band intensities were analyzed using gray values in Image J software. Data are shown as mean ± SD (analysis of variance). *P < 0.05 vs. SCI group, ***P < 0.001 vs. SCI group

Autophagy is an evolutionarily conserved lysosome-mediated catabolic pathway that ensures the degradation of dysfunctional cellular components to maintain homeostasis in response to various forms of stress. Several studies have shown that recovery from SCI can be facilitated by induction of autophagy [8, 29]. We observed the ultrastructure of rat spinal cord tissue 7 days after SCI using electron microscopy and found that the EF-sEVs group had more autophagosomes than the SCI group (Fig. 4A). Then, western blot revealed that the protein levels of Beclin1 and LC3II/LC3I were significantly upregulated, whereas p62 was downregulated, in the EF-sEVs group (Fig. 4B). Meanwhile, western blot of apoptosis-related marker proteins showed that apoptosis was significantly more inhibited in the EF-sEVs group than in the SCI group (Fig. 4C). Furthermore, western blot demonstrated that the SIRT1/AMPK signaling pathway, which can regulate autophagy, was activated (Fig. 4D). These results confirmed that EF-sEVs could induce autophagy and inhibit apoptosis in spinal cord tissue via the SIRT1/AMPK signaling pathway.

### EF-sEVs attenuated H_2_O_2_-induced neuronal apoptosis by activating autophagy in vitro

**Fig. 5:**
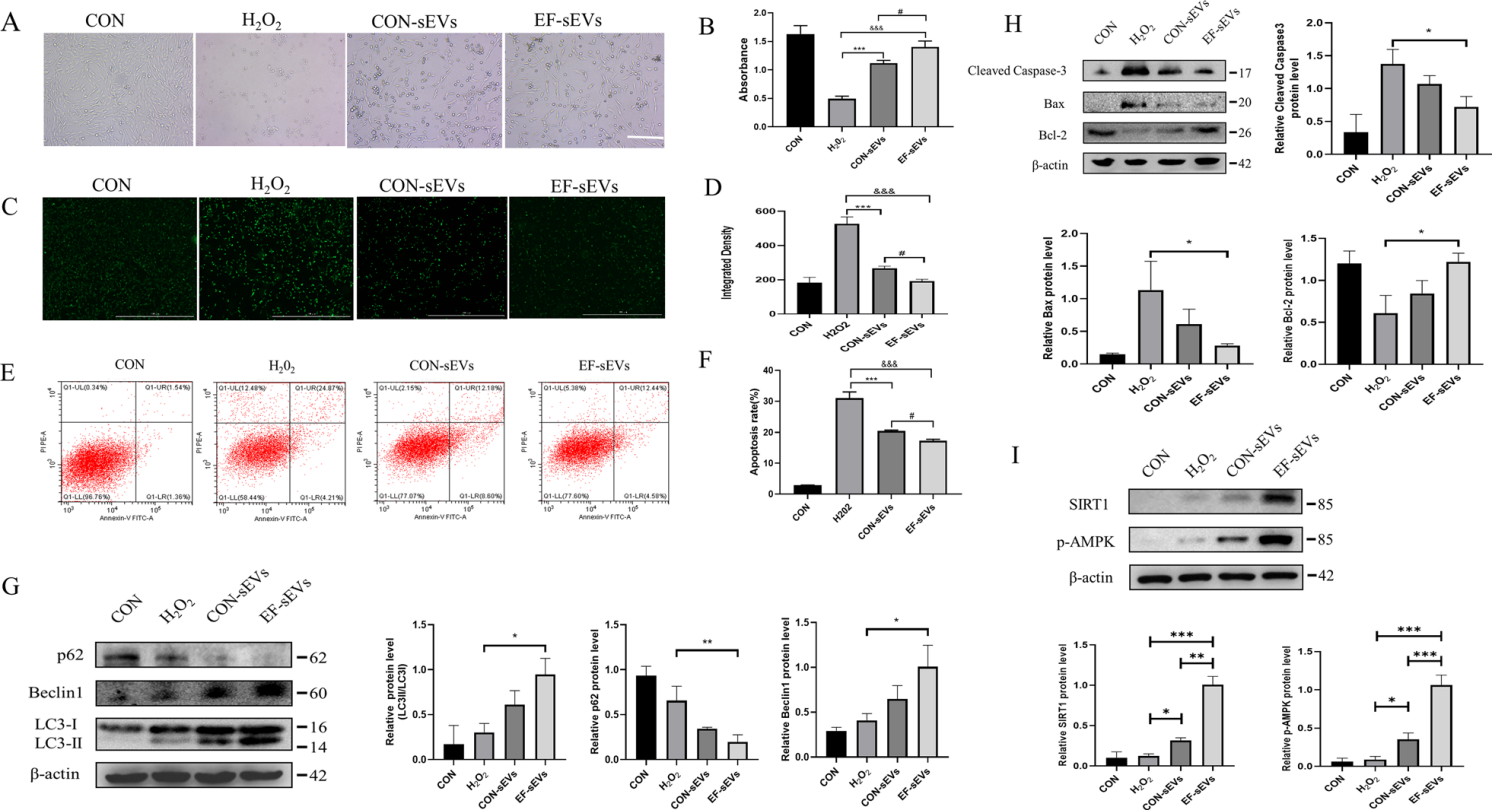
EF-sEVs attenuated H_2_O_2_-induced neuronal apoptosis by activating autophagy in vitro. **A** PC12 cell morphology changes under ×40 magnification after treatment with EF-sEVs and H_2_O_2_. **B** The protective effects of EF-sEVs on the viability of H_2_O_2_-exposed PC12 cells. **C** Representative images showing levels of ROS in each group. Scale bar=1000 μm. **D** Quantitative analysis of levels of ROS in PC12 cells in each group. **E** Apoptosis rates of PC12 cells determined by flow cytometry. **F** Percentages of apoptotic cells. **G** Western blot analysis of autophagy-related proteins levels and quantitative analysis results. **H** Western blot analysis of apoptosis-related proteins levels and quantitative analysis results. **I** Western blot analysis of SIRT1 and p-AMPK protein expression levels and quantitative analysis results. Data expressed as mean ± SD (n = 3)

Cellular oxidative stress is an important pathological change in the spinal cord after SCI. The H_2_O_2_-induced oxidative injury PC12 cell model is often used in in vitro studies of SCI. We observed morphological changes in cells after co-treatment with EF-sEVs or CON-sEVs and 200 µM H_2_O_2_. PC12 cells in the CON group adhered to the walls and grew normally, with a full shape and a long spindle shape, whereas the cells treated with 200 µM H_2_O_2_ showed shrinkage and a round shape, with a large number of dead cells floating in the culture medium. However, the morphological changes in cells treated with EF-sEVs and CON-sEVs were significantly improved (Fig. 5A). To study the effects of EF-sEVs and H_2_O_2_ on PC12 cells, we next used a CCK-8 kit to determine cell viability. As shown in Fig. 5B, and EF-sEVs and CON-sEVs treatment significantly improved the survival rate of PC12 cells damaged by H_2_O_2_. Compared with that of the CON-sEVs group, cell viability was significantly increased in the EF-sEVs group. Moreover, to investigate whether the protective effect of EF-sEVs on H_2_O_2_-stimulated PC12 cells was related to oxidative stress, we evaluated levels of intracellular ROS. Intracellular ROS level increased significantly after H_2_O_2_ stimulation, whereas ROS levels decreased after treatment with EF-sEVs (Fig. 5C, D). Furthermore, Annexin V-FITC/PI double staining and flow cytometry indicated that EF-sEVs had the strongest inhibition of apoptosis (Fig. 5E, F). Western blot also showed similar results (Fig. 5H). In addition, the autophagy flux of the EF-sEVs-treated injured PC12 cells was also detected by western blot. Autophagy was triggered by injury in neural cells, which could be enhanced by EF-sEVs treatment (Fig. 5G). The SIRT1/AMPK pathway is a key survival-signaling pathway involved in modulating autophagy [30]. To study whether the SIRT1/AMPK signaling pathway plays a role in H_2_O_2_-induced injury and the protective effect of EF-sEVs, we detected the expression of key proteins SIRT1 and p-AMPK using western blot. SIRT1 and p-AMPK levels were significantly higher in the EF-sEVs group than in the H_2_O_2_ group (Fig. 5I). This indicated that EF-sEVs participated in autophagy progression by regulating the SIRT1/AMPK signaling pathway.

### Blocking autophagic flux abrogates the anti-apoptosis and antioxidation effects of EF-sEVs in vitro

**Fig. 6:**
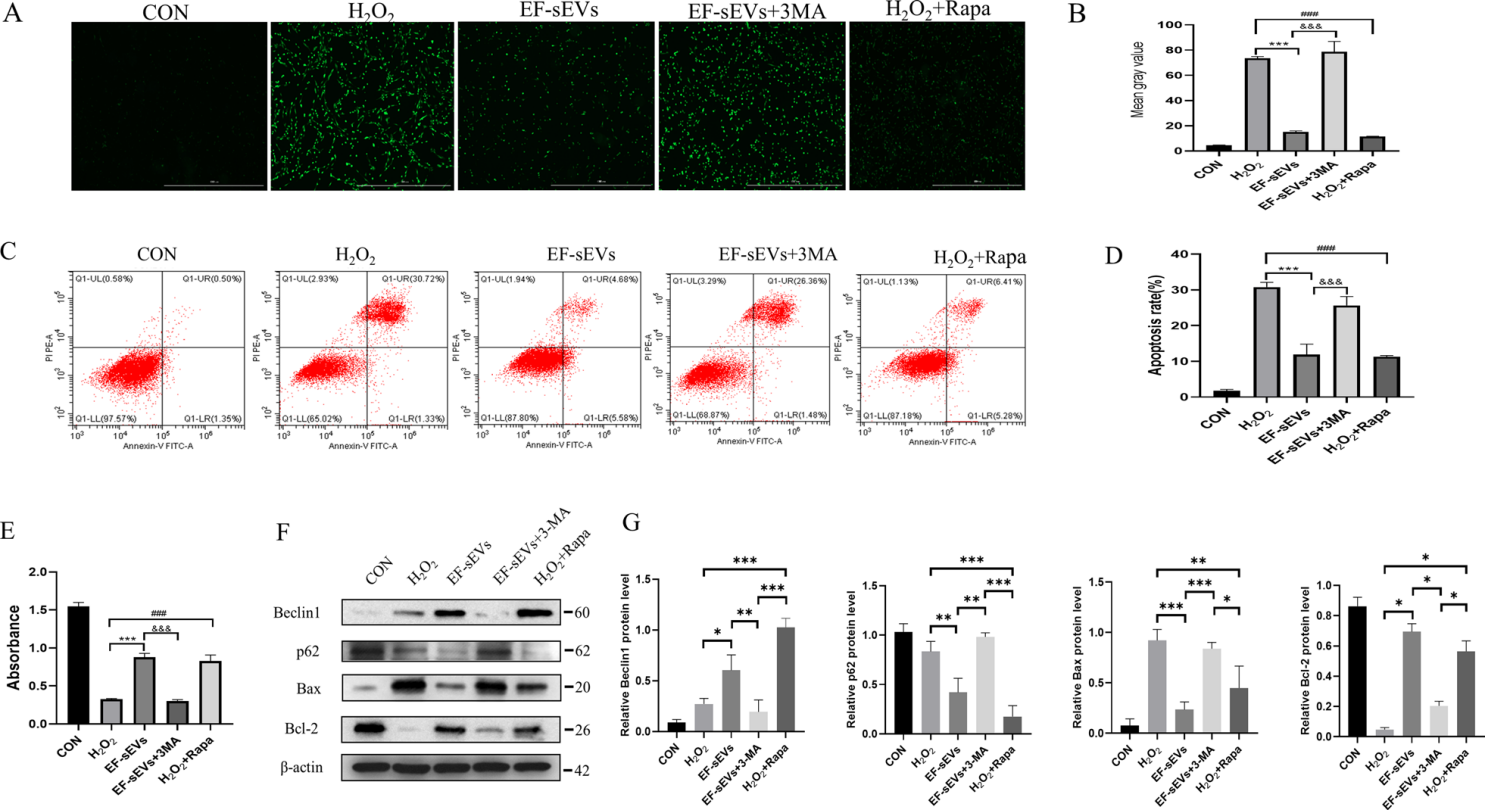
Blocking autophagic flux reversed the anti-apoptosis and antioxidation effects of EF-sEVs in vitro. **A** Representative images showing the levels of ROS in each group. Scale bar=1000 μm. **B** Quantitative analysis of levels of ROS in PC12 cells in each group. **C** The apoptosis rates of PC12 cells were determined using flow cytometry. **D** Percentages of apoptotic cells in each group. **E** The viability of H_2_O_2_-exposed PC12 cells were detected using CCK-8 assays. **F** Western blot analysis of autophagy-related and apoptosis-related proteins. **G** Quantitative analysis of autophagy-related and apoptosis-related protein expression levels. Data are expressed as mean ± SD (n = 3)

Because the results from previous studies and the aforementioned experiments implied that autophagy plays a key role in neuronal apoptosis after SCI, we hypothesized that the inhibition of apoptosis after EF-sEVs treatment is dependent on the activation of autophagy. To verify the relationship between autophagy and apoptosis, we used 3-methyladenine (3-MA), an inhibitor of autophagy, and rapamycin, an activator of autophagy. Consistent with the previous results, EF-sEVs still inhibited oxidative stress and promoted cellular vitality as shown by the ROS and CCK-8 assays, but addition of 3-MA disrupted these protective effects, and rapamycin restored them (Fig. 6A, B, E). Annexin V-FITC/PI double staining and flow cytometry also showed similar results (Fig. 6C, D). Western blot also showed that 3-MA inhibited the EF-sEVs-induced increase in the autophagy marker protein Beclin1 and the antiapoptotic protein Bcl-2. In addition, 3-MA reversed the reduction in p62 and Bax expression observed after EF-sEVs treatment (Fig. 6F, G). Taken together, these results suggested that autophagy activation induced by EF-sEVs was necessary for the inhibition of apoptosis.

### LncRNA-MALAT1 was a key component of EF-sEVs-induced neuroprotection

**Fig. 7:**
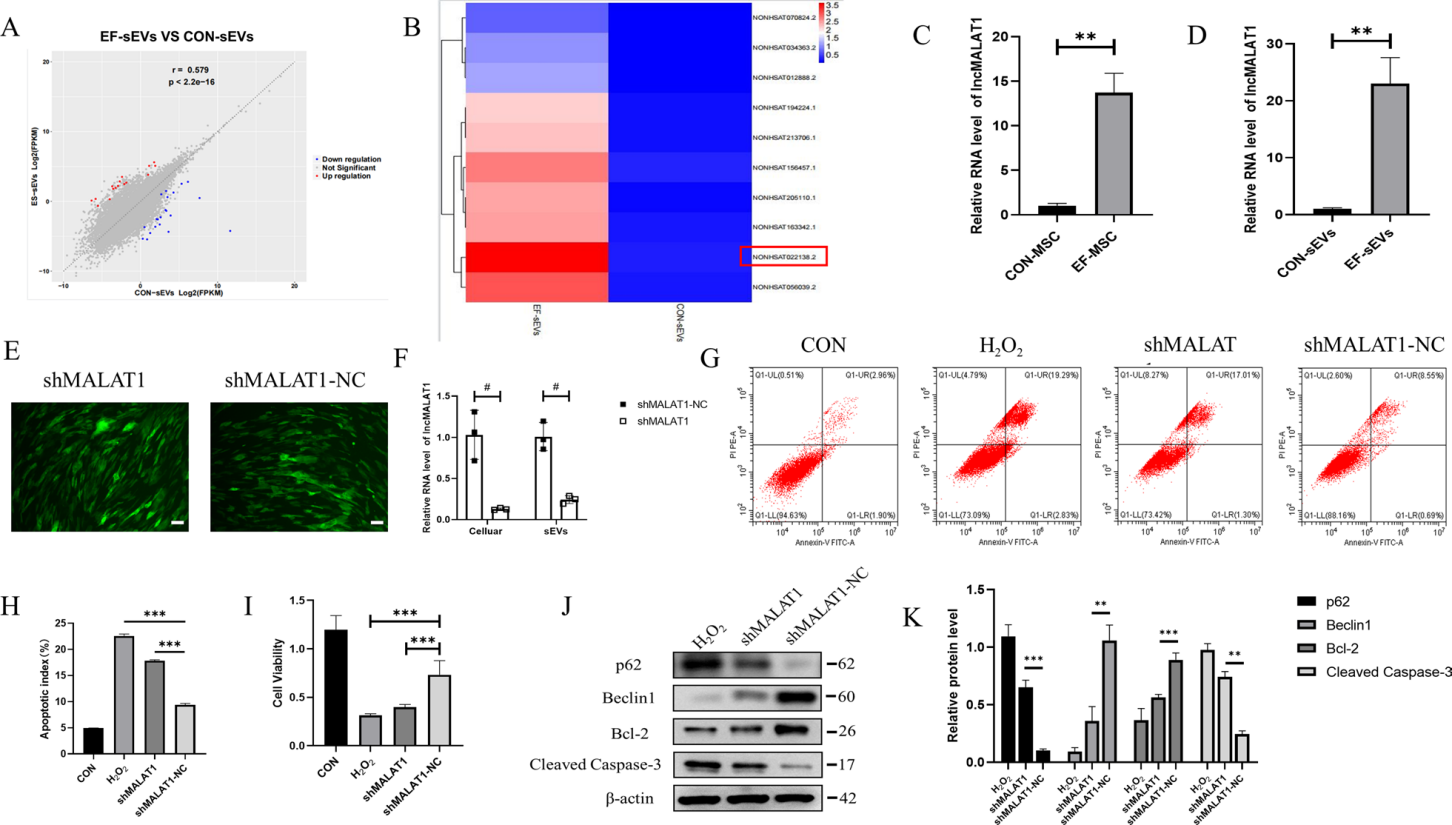
LncRNA-MALAT1 was a key component of EF-sEVs-induced neuroprotection. **A** Volcano plots of the results of RNA sequencing and differential lncRNA expression profile analysis. Blue indicates decreased expression and red indicates increased expression of the dysregulated lncRNAs in EF-sEVs (*p* < 0.05). The gray indicates no significant change. **B** Heatmap of differentially expressed lncRNAs. The top 10 upregulated lncRNA, lncRNA-MALAT1 (red box) increases most significantly. **C**, **D** Quantitative real-time PCR of lncRNA-MALAT1 in normal and EF condition in hucMSCs and sEVs, respectively. **E** Representative images show the hucMSCs transfected with shRNA or shRNA negative control (green). Scale bar: 100 μm. **F** qRT-PCR for lncRNA-MALAT1 in hucMSCs and sEVs after transfection. **G**, **H** Flow cytometry analysis of apoptosis percentage in PC12 cells treated with shRNA-EF-sEVs. **I** CCK-8 assay cell viability of H_2_O_2_-induced PC12 cells co-cultured with PBS, shMALAT1-EF-sEVs, and shMALAT1-NC-EF-sEVs. **J**, **K** Protein expression levels of autophagy-related proteins and apoptosis-related proteins, presented as the average expression normalized to β-actin levels. Data are expressed as mean ± SD (n = 3)

The results of in vitro and in vivo experiments showed that EF-sEVs had better effects compared with CON-sEVs in promoting behavioral recovery and neuroprotection. We hypothesized that the difference in lncRNA contents between EF-sEVs and CON-sEVs could be a major cause of this. Thus, we performed RNA sequencing and differential lncRNA expression profile analysis (Fig. 7A). According to the results of experiments in which we analyzed the top 10 upregulated factors, lncRNA-MALAT1 caused significant upregulation (Fig. 7B). To validate the sequencing results of lncRNA-MALAT1, we used qPCR to detect levels of lncRNA-MALAT1, and they were significantly elevated in MSCs and sEVs cultured in EF conditions (Fig. 7C, D). To further investigate the role of lncRNA-MALAT1, we used a lentivirus-based approach to knock down lncRNA-MALAT1 in hucMSCs. Then, shMALAT1 or a shMALAT1 negative control was transfected into EF-hucMSCs, leading to the production of green fluorescence (Fig. 7E). Next, sEVs were isolated. We used qPCR to confirm that lncRNA-MALAT1 levels were significantly reduced in cells and sEVs (Fig. 7F), which indicated successful transfection. The shMALAT1-EF-sEVs and shMALAT1-NC-EF-sEVs were both co-cultured with PC12 cells under H_2_O_2_ conditions. The results revealed that knockdown of lncRNA-MALAT1 attenuated the neuroprotective effects of EF-sEVs (Fig. 7G–I). Western blot showed that the effects of shMALAT1 in promoting autophagy to inhibit apoptosis were diminished (Fig. 7J, K).

### LncRNA-MALAT1 exerted neuroprotection effects by sponging miR-22-3p

**Fig. 8:**
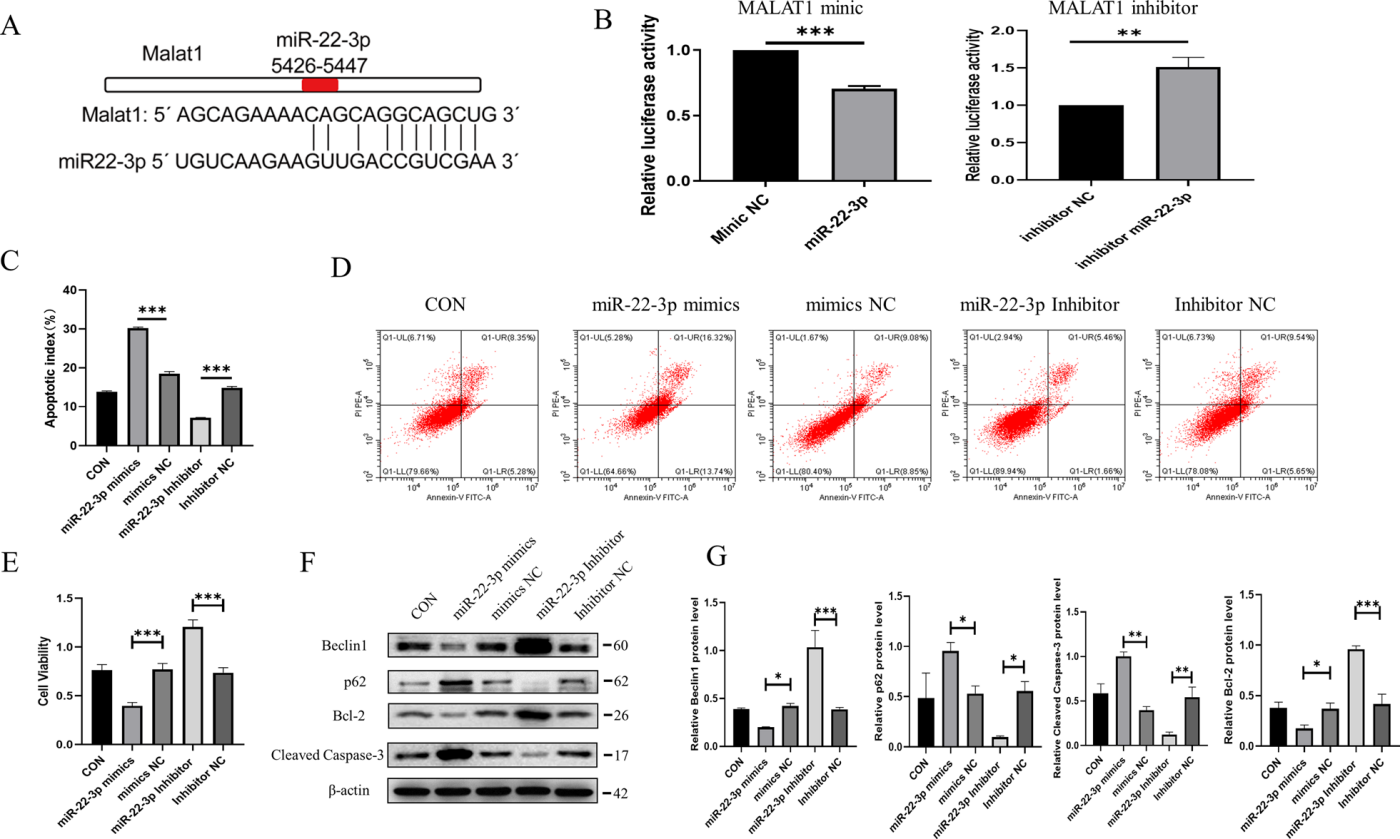
LncRNA-MALAT1 exerted neuroprotective effects by sponging miR-22-3p. **A** The binding site of lncRNA-MALAT1 and miR-22-3p. **B** Luciferase activity assay of HEK293T cells transfected with luciferase constructs containing mimics and inhibitor of lncRNA-MALAT1. **C**–**E** H_2_O_2_-induced PC12 cells were transfected with miR-22-3p mimics, miR-22-3p inhibitor, and their negative controls. Cell viability was assessed using CCK-8 assay (**C**). Cellular apoptosis was analyzed using flow cytometry (**D**, **E**). **F**, **G** Protein expression levels of Bcl-2, cleaved caspase 3, Beclin1, and p62 from H_2_O_2_-induced PC12 cells transfected with miR-22-3p mimics, miR-22-3p inhibitor, and their negative controls. Data are expressed as mean ± SD (n = 3)

We cloned lncRNA-MALAT1, with the putative miR-22-3p-binding sites, into a reporter plasmid and assessed responsiveness to miR-22-3p in HEK293T cells. The results of a dual luciferase assay demonstrated the binding site between lncRNA-MALAT1 and miR-22-3p (Fig. 8A, B). PC12 cells under H_2_O_2_ were then transfected with miR-22-3p mimics and inhibitors. The miR-22-3p inhibitors notably promoted neuroprotective effects, whereas miR-22-3p mimics attenuated these effects (Fig. 8C–E). miR-22-3p inhibitors downregulated cleaved caspase 3 and p62 expression while upregulating Bcl-2 and Beclin1 expression (Fig. 8F, G). These results suggested that lncRNA-MALAT1 contains a functional miR-22-3p binding site and sponges miR-22-3p.

### MiR-22-3p enhanced phosphorylation of AMPK in PC12 cells by targeting SIRT1

**Fig. 9:**
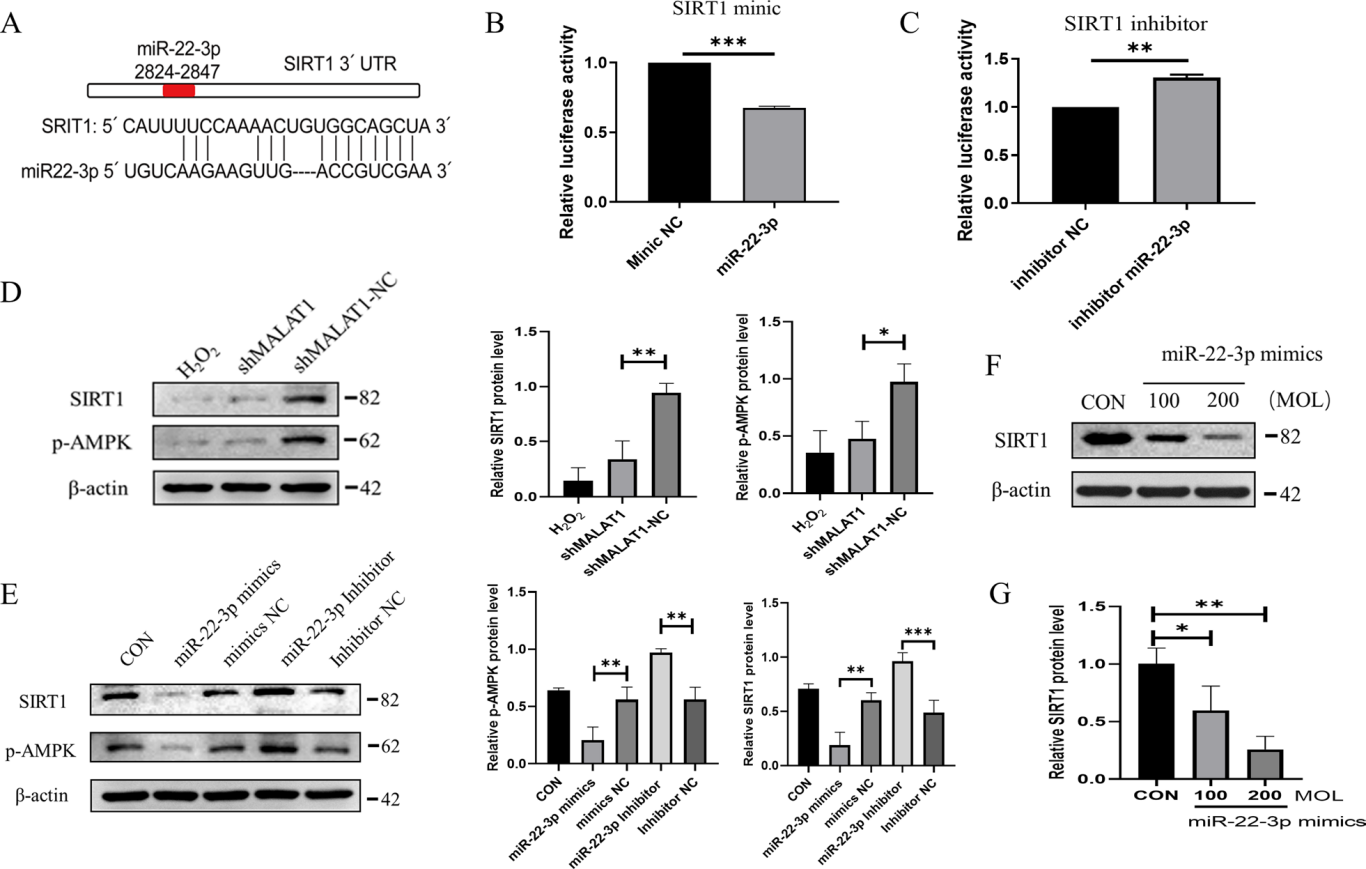
miR-22-3p enhanced AMPK phosphorylation in PC12 cells by targeting SIRT1. **A** The binding site of miR-22-3p and SIRT. **B**, **C** Luciferase activity assay of HEK293T cells transfected with luciferase constructs containing mimics and inhibitor of SIRT1. **D** Levels of SIRT1 and phosphorylated AMPK in H_2_O_2_-induced PC12 cells treated with shMALAT1-EF-sEVs and shMALAT1-NC-EF-sEVs. **E** SIRT1 and phosphorylated AMPK levels in PC12 cells treated with miR-22-3p mimics, miR-22-3p inhibitor, and their negative controls. **F**, **G** SIRT1 levels in H_2_O_2_-induced PC12 cells treated with different miR-22-3p versions in different concentrations

**Fig. 10:**
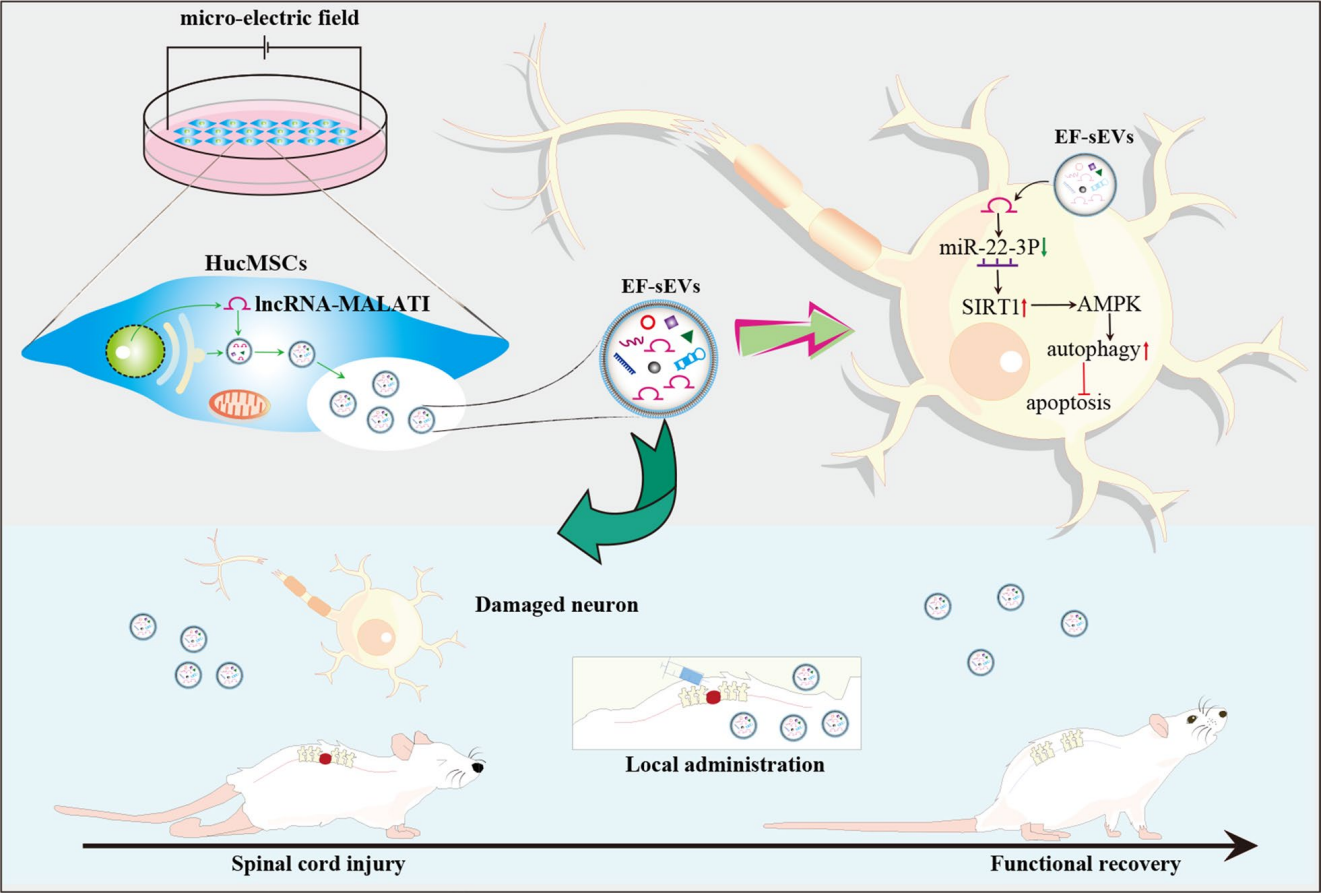
Schematic representation of the regulatory role of the EF-sEVs lncRNA-MALAT1 in SCI via mediation of the miR-22-3p/SIRT1/AMPK axis. HucMSCs induced by microelectric fields increase the expression of lncRNA-MALAT1, which in turn is encapsulated into EF-sEVs. EF-sEVs delivered lncRNA-MALAT1 into neurons. LncRNA-MALAT1 sponges adsorbed miRNA-22-3p and promotes the SIRT1/AMPK signaling pathway, which induced autophagy to inhibited apoptosis, thereby EF-sEVs improving the motor function in hindlimbs after SCI in rats

We predicted the target genes of miR-22-3p using the probability of interaction by target accessibility, miRmap, and Diana-microT tools. Among the possible miR-22-3p target genes that are also involved in autophagy pathways, we focused on SIRT1 (Fig. 9A). The results of a dual luciferase assay showed a significant decrease in SIRT1 mimic luciferase activity in HEK293T cells transfected with miR-22-3p, demonstrating an interaction between miR-22-3p and SIRT1 (Fig. 9B). Western blot showed that silencing lncRNA-MALAT1 lowered protein levels of SIRT1 and phosphorylation of AMPK (Fig. 9D). In addition, miR-22-3p mimics reduced the levels of phosphorylated AMPK and SIRT1 (Fig. 9E), and miR-22-3p inhibitors reversed this effect. Furthermore, transfection with miR-22-3p mimics decreased SIRT1 levels in a concentration-dependent manner (Fig. 9F, G). These data suggested an essential role of the MALAT1/miR-22-3p/SIRT1/AMPK axis in neuroprotection. Furthermore, the mechanism of EF-sEVs therapy promoting SCI repair was illustrated in Fig. 10.

## Discussion

SCI is a central nervous system injury disease with a little reversibility, high incidence, and a high disability rate, which brings a large burden to patients, their families, and society, and currently consumes a large amount of medical resources [31]. SCI leads to oxidative stress, inflammatory responses, ischemia, and nerve cell apoptosis, significantly affecting patient prognosis. Therefore, it is urgent to identify a novel and effective apoptosis intervention for repairing injured spinal cord tissue. Recent studies have shown that EF has neuroprotective effects in SCI; however, the underlying mechanisms remain unclear. In the present study, we carried out a series of in vivo and in vitro experiments to investigate the potential role of hucMSC-derived sEVs induced by EF in the treatment of SCI and further confirmed the mechanisms involved. Collectively, our findings indicated that EF-sEVs enabled enhancement of autophagic flux via activation of the lncRNA-MALAT1/miRNA-22-3p/SIRT1/AMPK signaling pathway to alleviate neuronal apoptosis, finally improving the motor function in hindlimbs after SCI in rats.

Various forms of EF stimulation, such as epidural electrical stimulation, peripheral nerve stimulation, and functional electrical stimulation, have been applied to treat SCI and have achieved good results [32, 33]. However, despite the positive outcomes observed in clinical studies, the underlying cellular and molecular mechanisms driving these functional improvements remain largely unknown. In this study, we first employed EF to cultivate hucMSCs and subsequently extracted the secreted sEVs. This innovative approach allowed us to create an optimal environment for the growth and proliferation of hucMSCs and to promote the release of some factors, including sEVs. These sEVs are known to contain various bioactive molecules such as proteins, lipids, and nucleic acids that play crucial roles in intercellular communication and tissue regeneration. Then, we investigated the effects and potential mechanisms of MSC-derived sEVs induced by EF on SCI in rats, aiming to gain insight into the sEVs content and potential therapeutic applications.

First, we established a rat model of SCI that simulated human spinal cord injury incurred by vertical impact with heavy objects. The systemic administration of sEVs is associated with delayed and inefficient delivery to the site of injury, suggesting that high doses are needed to reach therapeutic levels locally. Here we injected EF-sEVs directly at the lesion site acutely after SCI, as reported previously [34, 35]. Our preliminary in vivo studies demonstrated that the intralesional application of EF-sEVs alleviated SCI and promoted motor recovery in the hindlimbs of rats, and the repair effects were better than those seen in a CON-sEVs group.

Numerous studies have shown that apoptosis plays a pivotal role in SCI, and inhibiting apoptosis can be beneficial to the improvement of the motor function of hindlimbs [36–38]. The down regulation of the Bax/Bcl-2 ratio is associated with suppressed apoptosis [39]. Cleaved caspase 3 is also assumed to be a marker of and the main player in apoptosis [40]. After neuronal injury, the expression of proapoptotic proteins Bax and cleaved caspase 3 is upregulated, whereas the expression of antiapoptotic Bcl-2 is generally downregulated. It was reported that sEVs could exert antiapoptotic effects, which could provide protection from SCI [41]. In the present study, the ratio of Bax/Bcl-2 and the level of cleaved caspase 3 remarkably increased after SCI. However, they significantly decreased in the EF-sEVs group compared with the SCI group. This revealed that apoptosis was involved in SCI, and EF-sEVs alleviated neuronal apoptosis after acute SCI in rats. In vitro flow cytometry and western blot showed that pretreatment with EF-sEVs reduced apoptosis of neuronal PC12 cells subjected to H_2_O_2_-induced oxidative damage. Specifically, western blot showed that expression levels of proapoptotic markers were downregulated and that expression of the antiapoptotic marker Bcl-2 was upregulated.

In recent years, more and more attention has been paid to the critical roles of autophagy in SCI. Vast evidence has been accumulated showing that autophagy contributes to the maintenance of cellular homeostasis and to the quality control of proteins and subcellular organelles. Pathological conditions or cellular stress can induce autophagy as an adaptive and protective mechanism [42]. This autophagy flux has also been determined using autophagic markers LC3, proteins composing the autophagosome membranes, p62, proteins facilitating the degradation of autophagosomes and Beclin-1, an essential protein at the beginning of autophagy [10]. Whether enhanced levels of autophagy are beneficial or detrimental to neural cell maintenance during SCI repair depends on the context. For instance, previous studies have confirmed that triptolide significantly improved motor function in rats with acute traumatic SCI by promoting autophagy and inhibiting apoptosis [11]. Conversely, increased autophagic flux may lead to neuronal cell death through excessive autophagy (autophagic death) or through activation of apoptosis and other cellular death mechanisms [43]. Chen et al. found that excessive autophagy in SCI could lead to autophagic death in neurons, thereby affecting the regeneration of axons and the recovery of nerve function [44]. Therefore, the protective mechanisms of autophagy in SCI needed to be further investigated. Recent studies have reported that sEVs, in concert with the autophagy–lysosomal pathway, maintain intracellular protein and RNA homeostasis [45]. However, whether EF-sEVs can activate target cell autophagy to prevent tissue damage has not been reported. In the present study, we found that the ratio of LC3-II/I and p62 increased in the SCI group compared with the sham group. This means that the accumulated autophagosome increased, whereas its degradation slowed down, implying that autophagic flux was obstructed. However, we found that the ratio of LC3-II/I continued to increase but p62 levels decreased after treatment with EF-sEVs. Western blot of autophagy marker proteins from in vitro experiments further confirmed this. These results strongly suggested that EF-sEVs increased autophagy after SCI, suggesting a protective function of autophagy in neuronal recovery. To assess whether EF-sEVs-mediated autophagy is critical for inhibition of apoptosis and antioxidation, we co-treated PC12 cells with the autophagy-specific inhibitor 3-MA and observed a reversal of both the antiapoptotic and antioxidant effects of EF-sEVs.

RNA (including messenger RNA, miRNA, circular RNA, and lncRNA) and proteins are known to be highly concentrated in sEVs [46]. lncRNAs have been regarded as one of the main functional factors in sEVs. Several studies have reported that sEVs derived from MSCs exert their biological functions on target cells by transporting specific lncRNAs [47, 48]. It has been found that the MSC-derived sEVs lncRNA KLF3-AS1 is involved in sEVs-mediated chondrocyte proliferation induction and chondrocyte apoptosis inhibition via the miR-206/GIT1 axis [49]. However, whether EF-sEVs enhance autophagic flux after SCI with respect to regulatory mechanisms involving lncRNAs remains unknown. To further understand the regulatory mechanisms, we performed differential lncRNA expression profile analysis using the sequencing results of CON-sEVs and EF-sEVs, screening out lncRNA MALAT1, the molecule with the highest-fold change in terms of expression levels. Furthermore, lncRNA MALAT1 was transferred efficiently to target neuronal cells following treatment with EF-sEVs. Thus, EF-sEVs are clearly enriched with lncRNA MALAT1, which likely contributes to the biological effects of EF-sEVs. Then, we showed that the knockdown of lncRNA MALAT1 abolished the favorable anti-apoptosis and antioxidant effects of EF-sEVs. Thus, we provided evidence indicating that lncRNA-MALAT1 is shuttled during the EF-sEVs-mediated regulation of autophagy and apoptosis by targeting the miRNA-22-3p/SIRT1/AMPK axis. We speculate that EF-cultured hucMSCs promoted the transfer of lncRNA-MALAT1 into sEVs to mediate the activation of autophagy. The present results were consistent with this hypothesis. EF promoted the secretion of lncRNA-MALAT1-enriched sEVs from hucMSCs, which played neuroprotective roles in SCI. It has been reported that lncRNA-MALAT1 inhibited SCI-induced apoptosis by downregulating miR-204 [50], and the present findings were consistent with this role of MALAT1 in SCI. In addition, according to the sequencing results, it is possible that other significantly up-regulated lncRNAs in TOP 10 may have similar synergistic regulatory roles. However, among the up-regulated lncRNAs in TOP 10, except for lncRNA MALAT1 (TOP1) and lncRNA-SNHG16 (TOP 9), the other 8 are all novel lncRNAs. Those LncRNAs have not been fully studied, their functions are unclear, and have not been reported in the literature to date. This may be worth further investigation in our future work. As for lncRNA-SNHG16, it has been reported that it can repair sciatic nerve injury by promoting Schwann cell proliferation and migration [51]. But the role of lncRNA-SNHG16 in the central nervous system injury has not been studied, and the degree of upregulation is relatively low in our sequencing data, so we do not believe that it plays a major role in the present study. However, lncRNA-SNHG16 still has the potential for further research in SCI repair.

This study had certain limitations that should be considered. First, although intralesional injection is an efficient means by which to accurately deliver therapeutic genes to a target area, this strategy causes damage and the injected sEVs may leak at the injection site. Second, primary spinal cord neurons are a more preferable source for in vitro studies. Nonetheless, it has also been reported that PC12 cells are highly similar to primary spinal cord neurons in morphology, properties, and physiological functions [28]. The PC12 cell line is commonly used in research on neuroprotection, neurotoxicity, and neuro-inflammation. The PC12 cell model of oxidative stress induced by H_2_O_2_ is often used in in vitro studies of nervous system diseases. Third, SIRT1/AMPK signaling pathway inhibitors or their components were not used in vivo in this study. Thus, the detailed molecular mechanisms in vivo need to be further studied. Finally, due to the lack of targeting, these sEVs may also be absorbed by other cell types in the spinal cord, leading to extra-target effects. Engineering to modify sEVs may be an effective way to address this issue.

## Conclusions

In summary, our findings demonstrated for the first time that EF promoted hucMSCs to secrete lncRNA-MALAT1-enriched sEVs that may play neuroprotective roles to effectively enhance spinal cord repair after SCI. Furthermore, we proved that EF-sEVs inhibited apoptosis by activating autophagy via the lncRNA-MALAT1/miR-22-3p/SIRT1/AMPK axis, which in turn exerted neuroprotective effects. This provides a promising strategy for the treatment of SCI and novel insights into the therapeutic roles of sEVs in EF therapy.

### Supplementary Information

**Additional file 1: Figure S1.** Schematic diagram of micro-electric field incubation device.** Figure S2.** Identification and characterization of hucMSCs.

**Additional file 2: Table S1.** Sequences of primers, miR-22-3p mimics, inhibitor and their negative controls. **Table S2.** The shRNA sequences list.

## Data Availability

The data supporting the conclusions of this article are included within the article and its Additional files.
