# Supplementary material for: Micro electrical fields induced MSC-sEVs attenuate neuronal cell apoptosis by activating autophagy via lncRNA MALAT1/miR-22-3p/SIRT1/AMPK axis in spinal cord injury
Source: J Nanobiotechnology. 2023 Nov 27;21:451. doi: 10.1186/s12951-023-02217-2 (PMC10680254; doi:10.1186/s12951-023-02217-2)
Supplement: Supplementary file 1 — Additional file 1: Figure S1. Schematic diagram of micro-electric field incubation device. Figure S2. Identification and characterization of hucMSCs. [file 12951_2023_2217_MOESM1_ESM.docx]

**
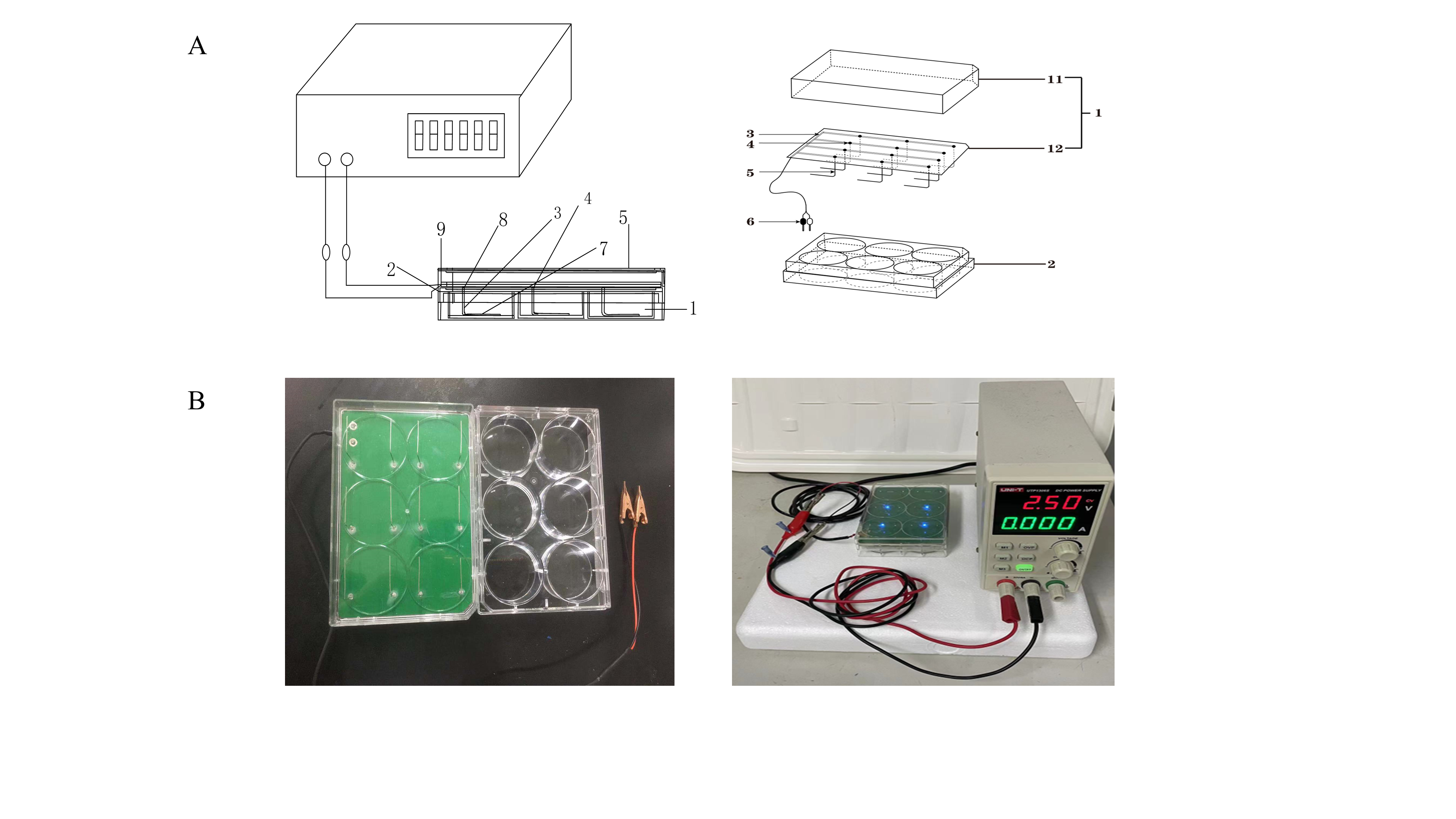
**

**Figure S1. Schematic diagram of micro-electric field incubation device.** (A) The overall schematic diagram of the micro-electric field culture device (Left) and the structural disassembly diagram of the culture part (Right), which is our own design and development and has been authorized by China Patent. (B) Physical drawing of the cell culture portion of the device (Left). Physical drawing of the complete unit in working condition (Right).


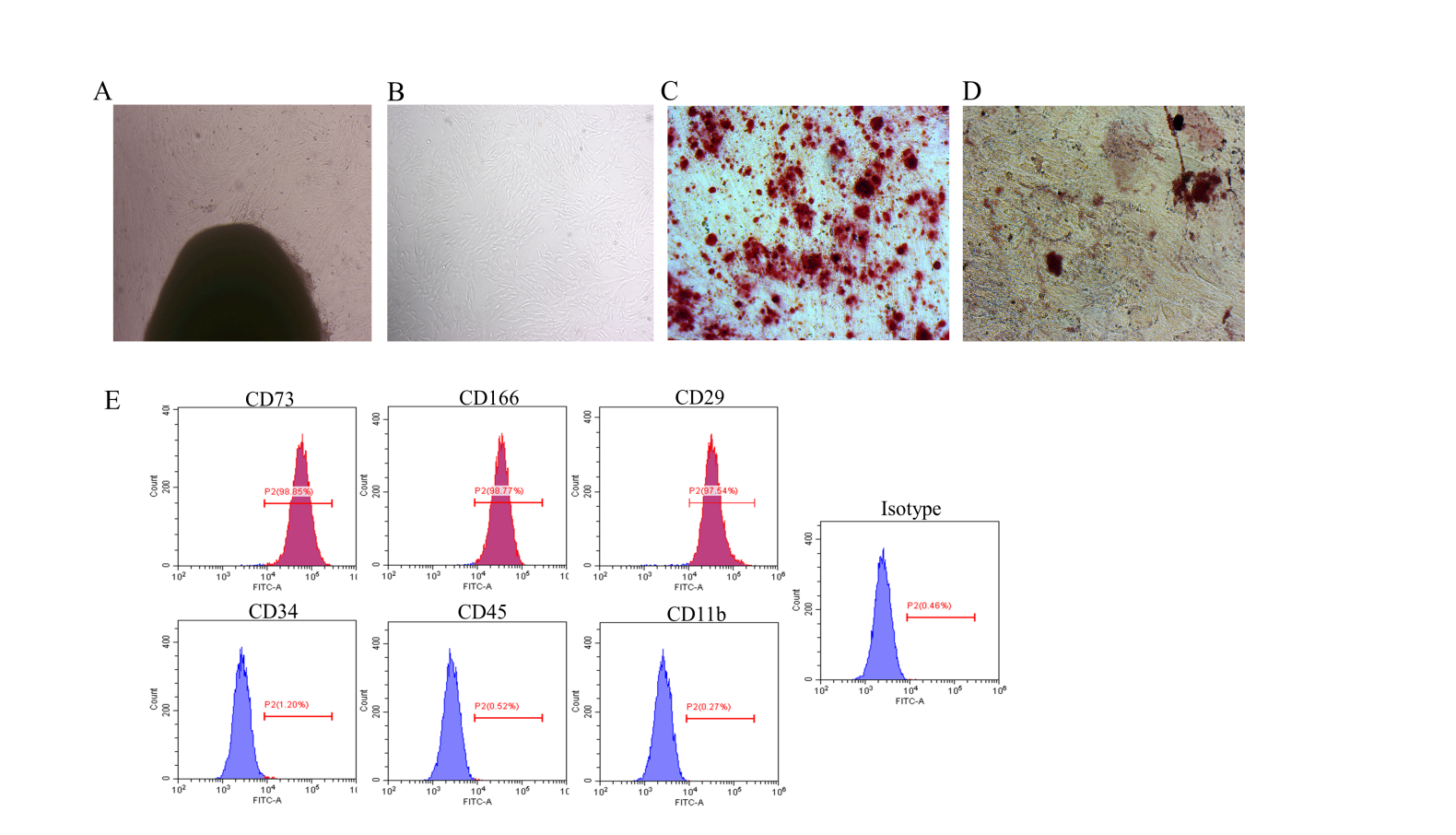


**Figure S2. Identification and characterization of hucMSCs.** (A) Primary human umbilical cord mesenchymal stem cells crawl out of umbilical cord tissue mass and adhere to the cell culture dish. (B) Morphological identification of hucMSCs (40✕). (C) Stem cell induced differentiation into adipocytes (100✕). (D) Stem cell induced differentiation into osteocytes (100✕). (E) Flow cytometry analyses of various phenotypic markers for hucMSCs.
