# Supplementary material for: Micro electrical fields induced MSC-sEVs attenuate neuronal cell apoptosis by activating autophagy via lncRNA MALAT1/miR-22-3p/SIRT1/AMPK axis in spinal cord injury
Source: J Nanobiotechnology. 2023 Nov 27;21:451. doi: 10.1186/s12951-023-02217-2 (PMC10680254; doi:10.1186/s12951-023-02217-2)
Supplement: Supplementary file 2 — Additional file 2: Table S1. Sequences of primers, miR-22-3p mimics, inhibitor and their negative controls. Table S2. The shRNA sequences list. [file 12951_2023_2217_MOESM2_ESM.docx]

**Table S1. Sequences of primers, miR-22-3p mimics, inhibitor and their negative controls.**

| **Primers** | **Sequences (5'-3')** |
| --- | --- |
| **Primers for qPCR** |  |
| MALAT1-F | TTACGGTTGGGATTGGTGGG |
| MALAT1-R | ACTGCCAGGCTGGTTATGAC |
| Human miR-22-3p-F | GCGGTCAAGCTGCCAGTT |
| Human miR-22-3p-R | TATGGTTGTTCACGACTCCTTCAC |
| GAPDH-F | TCAAGATCATCAGCAATGCC |
| GAPDH-R | CGATACCAAAGTTGTCATGGA |
| U6-F | CGCTTCGGCAGCACATATAC |
| U6-R | TTCACGAATTTGCGTGTCATC |
| **miR-22-3p mimics, inhibitor** |  |
| miR-22-3p mimics | AAGCUGCCAGUUGAAGAACUGU |
|  | AGUUCUUCAACUGGCAGCUUUU |
| miR-22-3p mimics negative control | UUCUCCGAACGUGUCACGUTT |
|  | ACGUGACACGUUCGGAGAATT |
| miR-22-3p mimics inhibitor | ACAGUUCUUCAACUGGCAGCUU |
| miR-22-3p mimics inhibitor negative control | CAGUACUUUUGUGUAGUACAA |

**Table S2.** **The shRNA sequences list**

| **Primers** | **Sequences (5'-3')** |
| --- | --- |
| **MALAT1 shRNA and negative control** |  |
| LV-UCA1-RNAi | CAGCCCGAGACTTCTGTAAAGGACT |
| LV-UCA1-RNAi negative control | CACAGGGAAAGCGAGUGGUUGGU |
